# Physical and Physiological Characteristics of Elite CrossFit Athletes

**DOI:** 10.3390/sports12060162

**Published:** 2024-06-12

**Authors:** Bram Sauvé, Magnus Haugan, Gøran Paulsen

**Affiliations:** 1 Department of Physical Performance, Norwegian School of Sport Sciences, Sognsveien 220, 0863 Oslo, Norway; magnushaugan@outlook.com (M.H.); goranp@nih.no (G.P.); 2 Norwegian Olympic Federation, Sognsveien 228, 0863 Oslo, Norway

**Keywords:** strength, aerobic, anaerobic, body composition, functional training, jump

## Abstract

Due to little available research on elite CrossFit athletes, the present investigation was undertaken to provide knowledge about their physical and physiological characteristics. Nineteen international level CrossFit athletes (8 men; 11 women) were tested for maximum oxygen consumption ($\dot{V}$O_2_max), Wingate, squat 1 repetition maximum (1RM), countermovement jump (CMJ), lower body force–velocity, and body composition (DXA). To provide perspective, the results were compared to sixteen elite-level Alpinists (8 men; 8 women). There was no significant difference in absolute nor relative $\dot{V}$O_2_max between the CrossFit and Alpinist men (*p* = 0.335 and *p* = 0.279, respectively). The CrossFit women showed similar absolute but higher relative $\dot{V}$O_2_max than the Alpinist women (*p* = 0.055 and *p* = 0.005, respectively). Mean anaerobic power was higher in both CrossFit men and women (*p* = 0.021 and *p* = 0.008, respectively). There were no significant differences in squat 1RM and CMJ for both men and women (*p* > 0.05). Both CrossFit men and women showed lesser lower body force production (*p* = 0.043 and *p* = 0.034, respectively) but higher power (*p* = 0.009 and *p* = 0.003, respectively). The CrossFit men and women had a lower fat mass (*p* = 0.018 and *p* = 0.002, respectively) and fat percentage (*p* = 0.027 and *p* < 0.001, respectively). These observations show that elite CrossFit athletes possess physical and physiological characteristics comparable to world-class Alpinists.

## 1. Introduction

CrossFit is a functional fitness training method and sport aimed at improving work capacity (i.e., strength, endurance, power, and balance) by repeatedly performing a circuit or series of tasks via a wide range of intensities and time domains from various disciplines. These disciplines include powerlifting, weightlifting, gymnastics, and traditional aerobic exercise (e.g., running, cycling, and rowing) [1]. Performance in each task is assessed by measuring the time needed to complete the task in question, by the total weight lifted, or by the total number of repetitions performed during a so-called “Workout of the Day” [1]. For the sport of CrossFit, the best CrossFit athletes compete in the World Championship, known as the CrossFit Games, where the winner is awarded the title “the Fittest on Earth”. Performance in CrossFit is a product of physiological, psychological, and technical factors such as strength, aerobic and anaerobic capacity, endurance, sport-specific technique, as well as mental strength. In this sport, unlike many other sports, athletes should not be specialized in a single form of training [2,3]. Since its origin in 2000, CrossFit has grown exponentially in popularity, and it is therefore of great importance to acquire knowledge about this sport.

Physiological aspects of CrossFit have previously been assessed by measurements of aerobic and anaerobic capacity, absolute and relative strength, power in the lower limbs, as well as body composition [4,5,6,7,8,9,10,11,12,13]. Some studies have examined the differences in physical capacity between CrossFit athletes of different competition levels and further categorized physical capacities from normative data [5,9,14,15,16,17]. The results of these studies refer to CrossFit athletes (men and women, ≥18 years) with varying levels of strength, maximal oxygen uptake (~54 and ~44 mL·kg^−1^·min^−1^ for men and women, respectively), and anaerobic capacity (Wingate mean power: ~650 and ~440 W for men and women, respectively), which can be expected from trained individuals [4,5,6,7,8,9,10,14,16]. These results, however, are assumed to not be representative of those of elite athletes.

Despite a growing interest in CrossFit—now a worldwide sport—there is currently little available research regarding the physical capacity of elite CrossFit athletes. Therefore, the present investigation was undertaken to provide novel knowledge about the physical and physiological characteristics of this population. These findings could offer insights into the physical demands it requires of the respective athletes on the elite level, potentially leading to improved training regimens and a better identification of talent and sports success by practitioners.

Alpine skiing is a sport known for athletes that are especially well trained in both strength and endurance capabilities [18,19,20]. The Norwegian National Alpine skiing team has been among the most successful teams in the world for decades. Since the 1990s, the Norwegian training philosophy has been based on versatile training of physical capabilities, such as strength, aerobic and anaerobic endurance [18,19,20,21]. This is the basis of their slogan, “Jack of all trades, master of none”. This is close to the philosophy of CrossFit and Alpinists are, hence, an excellent standard against which to measure CrossFit athletes. We hypothesized that elite CrossFit athletes possess a physical capacity comparable to world-class Alpinists.

## 2. Materials and Methods

### 2.1. Study Design

This study is a descriptive cross-sectional study of elite CrossFit athletes. The athletes performed four physical tests in the form of maximum oxygen consumption, Wingate, countermovement jump, and lower body force–velocity, in addition to having their body composition measured. Squat 1 repetition maximum (1RM) was self-reported by each athlete. The tests were meant to provide an overview of the athletes’ physical and physiological characteristics.

### 2.2. Participants

The CrossFit athletes and Alpinists were given detailed information about the study when asked to participate. Nineteen CrossFit athletes (8 men and 11 women, CFM and CFW, respectively) active at a very high national, as well as international, level were recruited for this study (Table 1). Recruitment was opportunistic via approaching local CrossFit centers (Oslo, Norway) and via open platforms. To be able to participate in the study, the following inclusion criteria had to be met:

1. Men and women > 18 years.
2. Participated in or qualified for the CrossFit Games^1^.
3. Participated in the World Championship, Functional Fitness.
4. Participated in Regionals^2^/Sanctionals^3^.
5. National top 15, CrossFit Open 2019 and/or 2020.

^1^Due to COVID-19, some athletes who were eligible for the 2020 CrossFit Games lost their right to participate due to changes and severe restrictions.

^2^Regionals: Previous international competitions (2018) based on region, directed by CrossFit, LLC. (Europe, South America, Oceania, North America, Asia, Africa).

^3^Sanctionals: Independent international competitions held and organized by CrossFit, LLC.

#### Exclusion Criteria

Illness or injuries that made it impossible to carry out the study’s tests.

To be able to compare the results between CrossFit athletes and Alpinists, sixteen Alpinists (8 men and 8 women, AM and AW, respectively) at a similar level of competition were recruited in this study (Table 1). Recruitment was carried out by approaching the Norwegian Alpine National Team’s coaches. To participate in the project, athletes had to be on the Norwegian national team in Alpine skiing between 2018 and 2020. All skiers competed in the FIS Alpine Ski World Cup, including World and Olympic champions. The included tests were part of the Alpinists’ annual test battery. Data were collected from a database at the Norwegian Olympic Center (Oslo, Norway).

### 2.3. Procedures

**Figure 1:**
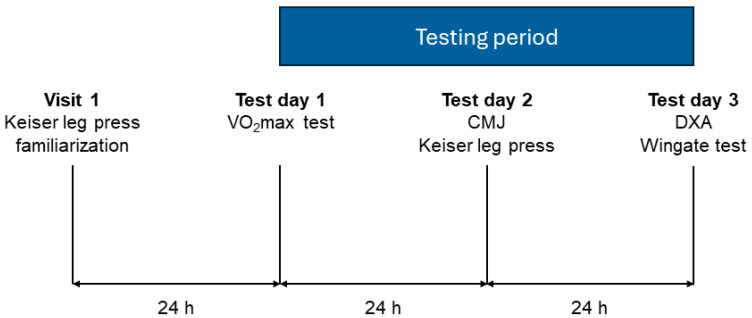
**Test protocol for the CrossFit athletes.** Abbreviations: $\dot{V}$O_2_max: maximum oxygen consumption. CMJ: countermovement jump. DXA: dual-energy X-ray absorptiometry.

To ensure that the CrossFit athletes showed up well recovered and that fatigue did not negatively affect the results, they were told to only perform light training 24 h prior to each test day. Sleep, food, and caffeine intake were not controlled for throughout the study’s test period. An overview of the test protocol for the CrossFit athletes can be seen in Figure 1.

### 2.4. Aerobic Capacity

**Figure 2:**
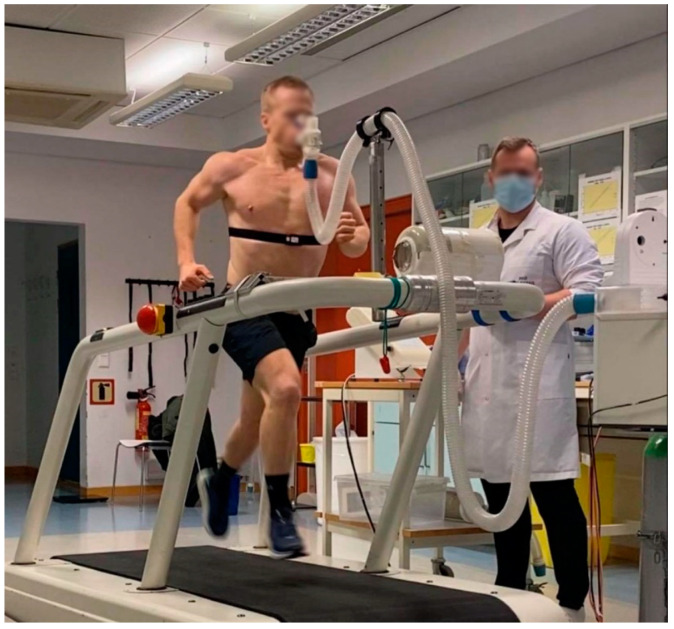
**A CrossFit athlete performing the maximum oxygen consumption test.** The aerobic capacity was recorded by means of a maximum oxygen uptake test. The athletes breathed through a two-way valve with a nozzle (Hans Rudolph Inc., Shawnee, KS, USA) while they ran on a treadmill (PPS 55 Sport Woodway Inc., Waukesha, WI, USA) at a 5.3% incline.

The ergospirometry system Oxycon Pro with mixing chamber (Jaeger Instr., Wuerzburg, Germany) was used to find the athletes’ maximum oxygen consumption ($\dot{V}$O_2_max). The gas analyzer was calibrated with certified calibration gases of known concentrations before every test. The volume meter was calibrated manually using a 3-L hand-held pump (Calibration Syringe, Series 5530, Hans Rudolph Inc., Shawnee, KS, USA). The athletes breathed through a two-way valve with a nozzle (Hans Rudolph Inc., Shawnee, KS, USA) while they ran on a Woodway treadmill (PPS 55 Sport Woodway Inc., Waukesha, WI, USA), as can be seen in Figure 2. The oxygen consumption ($\dot{V}$O_2_) was collected every 30 s.

The $\dot{V}$O_2_max test was conducted as a step protocol; from the start, the speed increased by 1 km·h^−1^ every minute. The athletes’ starting speed was 11 km·h^−1^ for the men and 9–11 km·h^−1^ for the women, with the treadmill at a 5.3%/3 degrees incline. If the athletes were no longer able to increase their speed, they maintained the same speed throughout the test until exhaustion and/or a plateau in $\dot{V}$O_2_ was reached. $\dot{V}$O_2_max was calculated as the average of the two highest consecutive 30 s $\dot{V}$O_2_ measurements.

### 2.5. Anaerobic Capacity

**Figure 3:**
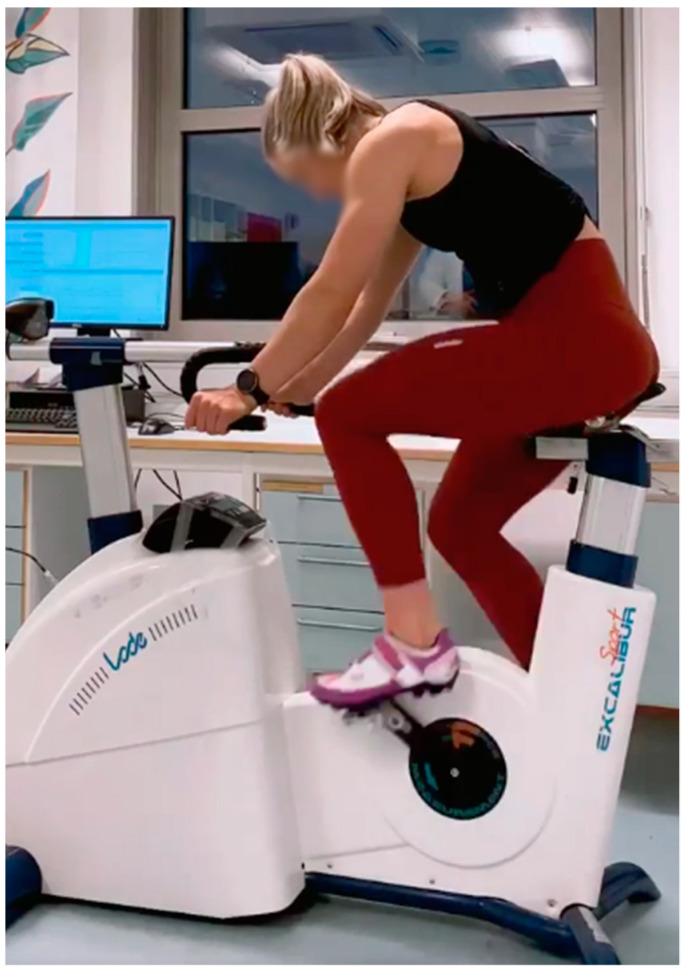
**A CrossFit athlete performing the Wingate test.** The anaerobic capacity was recorded via a 30-s Wingate test on a Lode Excalibur Sport (Lode B.V., Groningen, the Netherlands).

The CrossFit athletes conducted a 30 s Wingate test on a Lode Excalibur Sport (Lode B.V. Groningen, The Netherlands), as seen in Figure 3. The Alpinists performed the test on a Wattbike Pro (Wattbike, Nottingham, UK). The Lode Excalibur Sport records values every 200 ms, whereas the Wattbike Pro registers per pedal cycle. The power measurements of the Wattbike were validated against those of the Lode Excalibur Sport and showed no significant differences [22]. Each ergometer was adjusted according to the athlete’s preference for seat height, the horizontal distance between the tip of the seat and bottom bracket, and handlebar position.

Before the Wingate test, a 10 min general warm-up was performed at submaximal load (50–100 Watts) followed by three 6 s sprints. For the CrossFit athletes, the 6 s sprints were performed with three different braking resistances to individualize which braking resistance should be used during the Wingate test (CFW: 6 s 1: 0.5, 6 s 2: 0.6, 6 s 3: 0.7 Nm·kg^−1^; CFM: 6 s 1: 0.7, 6 s 2: 0.8, 6 s 3: 0.9 Nm·kg^−1^ being 5.1–7.1% and 7.1–9.2% of body mass for women and men, respectively). For the Alpinists, the 6 s sprints were performed with a different “Air Setting” based on the weight of the athlete to individualize the braking resistance. There was a 4 min break after the first and second sprints, followed by 5 min break after the last sprint before starting the 30 s Wingate test. The Wingate test started with the athletes pedaling at 60 revolutions per minute without braking resistance. After a 3 s countdown, consistent braking resistance was introduced to the flywheel and sustained throughout the following 30 s of the test. Lode Ergometry Manager 9.s3.1.0 (Lode B.V. Groningen, the Netherlands) and Wattbike Hub (Wattbike, Nottingham, UK) software were used to determine instantaneous power values (peak power and average power) on the respective ergometers. Average power was used as a measure of anaerobic capacity. During the test, strong verbal encouragement was given. The test was performed while sitting, and cycling shoes (Shimano pedals, PD-R7000) were used.

### 2.6. Jump Performance

**Figure 4:**
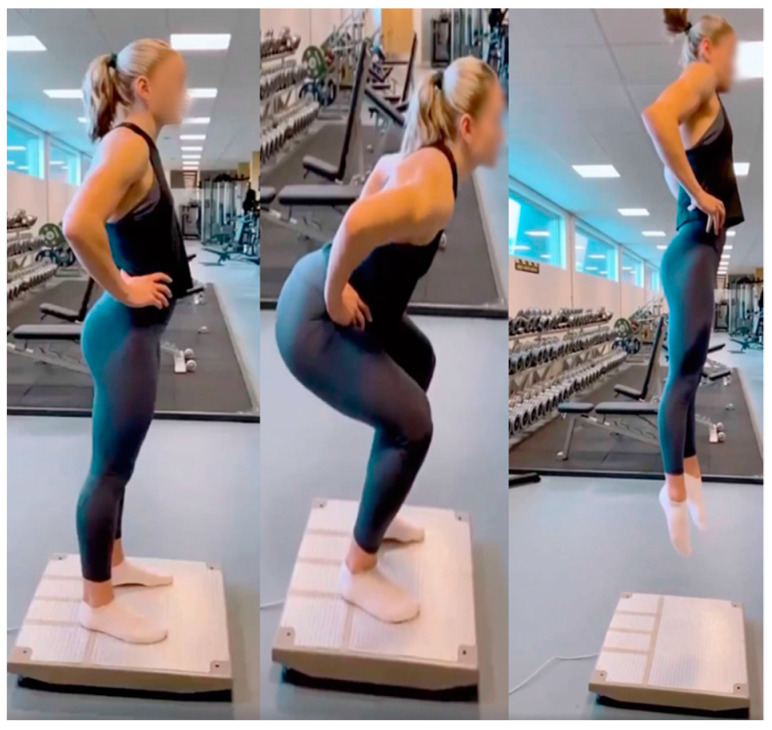
**A CrossFit athlete performing the countermovement jump (left to right: start position—depth—execution).** Jump performance was determined via the countermovement jump on a portable force platform (HUR Labs FP4, Tampere, Finland).

The CrossFit athletes performed the countermovement jump (CMJ) on a portable force platform (HUR Labs FP4, Tampere, Finland) with a sampling frequency of 1200 Hz, as seen in Figure 4. The Alpinists performed the CMJ on a floor-mounted power platform (AMTI, Advanced Mechanical Technology Inc., Watertown, WI, USA) with a sampling frequency of 2000 Hz. Software used for data acquisition was Force Platform Suite version 2.65.5.6 (HUR Labs, Tampere, Finland) and VALD Hub (VALD Performance, Queensland, Australia) for the CrossFit athletes and Alpinists, respectively. Force platforms are accepted as the gold standard when it comes to measuring jump height, validation of jump methods, and assessment of power development in athletes [23]. The force platform measures force in the vertical direction, and the force platforms’ software calculates jump height using the exit velocity (impulse–momentum method) [24]. A minimum of three jumps were completed, with a 30–60 s break between each jump. The athletes were encouraged to jump as high as possible. They jumped without shoes and with their hands on their hips (akimbo). If an attempt was invalid, or if the athlete continued to increase their jump height, more attempts were allowed. The best jump was used for further analysis.

### 2.7. Lower Body Force–Velocity (Keiser Leg Press)

**Figure 5:**
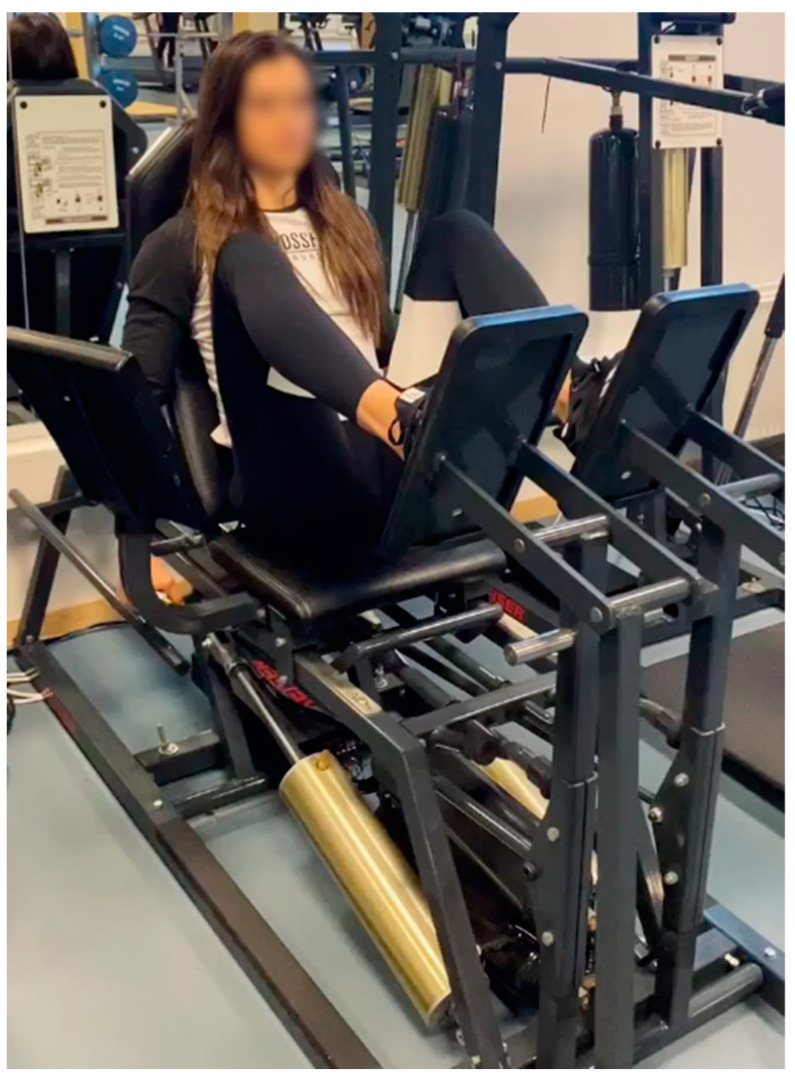
**A CrossFit athlete performing the Keiser leg press.** Lower body force, velocity, and power measurements were determined using a Keiser leg press (Keiser Pneumatic Leg Press Air 420, Keiser Corporation, Fresno, CA, USA). The picture shows the standardization of the femur (starting position).

Lower body force, velocity, and power measurements were carried out using a Keiser leg press (Keiser Pneumatic Leg Press Air 420, Keiser Corporation, Fresno, CA, USA). The Keiser leg press is a training device based on pneumatic resistance that has previously been shown to be highly reliable after investigations for test–retest reliability [25,26]. In this study, all actions from the right and left legs were performed simultaneously but unilaterally to detect differences between legs. A familiarization day was carried out to determine the standardized starting position (vertical femur in starting position; Figure 5) for each individual and to find the estimated 1RM that was used in the protocol during the test day. The leg press was performed with shoes. To find force, velocity, and power variables during the test day, a 10-step protocol built into Keiser’s software was performed. The 10-step protocol takes the estimated 1RM and divides it into 10 separate repetitions. After 10 min of general warm-up on an ergometer bike, two warm-up repetitions at 70% and 90% of the estimated 1RM were carried out. The 10-step protocol started at ~15% of the estimated 1RM, and the load was increased in fixed increments (20–30 kg) for each repetition until the estimated 1RM was reached at repetition 10. The pause between repetitions became progressively longer with increasing load (5–30 s). The force and velocity values across all repetitions were taken for each leg from the average development during the concentric phase. The eccentric phase was submaximal and not registered. The power results from each repetition were the product of force × velocity. The highest force, velocity, and power variables were defined as F_max_, V_max,_ and P_max,_, respectively. The athletes were verbally motivated to perform all repetitions with maximal intentional velocity.

### 2.8. Body Composition (DXA)

Body composition was determined using a dual-energy X-ray absorptiometry (DXA) scan (GE Lunar iDXA, GE HealthCare Technologies, Inc., Chicago, IL, USA). DXA has been shown to provide high precision when it comes to measuring bone mineral density, fat-free mass, and fat percentage if a well-standardized procedure is followed regarding activity and nutrition prior to the scan [27]. Quality assurance of the DXA scan was guaranteed by calibration prior to all scans to provide the highest measurement accuracy. The athletes were told not to perform hard exercises and to be rehydrated the day prior to the scan. The scan took place after they fasted for a minimum of 12 h. When the athletes arrived, their height and weight were recorded, and they removed any metal or jewelry. An entire body scan was taken following the company’s recommended subject positioning procedures and supplied algorithms. Total and regional bone mass, fat-free mass, and fat percentage were calculated after manual analysis and division in segments to distinguish between head, torso, and lower and upper extremities using GE, Lunar enCORE (software version 18).

### 2.9. Statistical Analysis

All data in text, figures, and tables are presented as mean ± standard deviation unless stated otherwise. Visual inspection and subsequent Shapiro–Wilk tests were performed for all data to confirm the normality of the distribution. To test for the differences between groups, independent Student’s t-tests were performed. Skewed data were examined using Mann–Whitney tests. Statistical significance was set at an alpha level of 0.05. Data processing was performed in Microsoft Excel for Microsoft 365 (Microsoft Corporation, Redmond, USA). Data analysis was performed in SPSS (version 27.0, IBM Corp., Armonk, NY, USA). Visualization of the data was performed in GraphPad Prism 9 (GraphPad Software Inc., La Jolla, CA, USA). The mean difference (∆) was calculated as ((mean CrossFit athletes—mean Alpinists)/mean Alpinists) × 100%. The strength of the difference was further explained by the effect size (ES) in the form of Hedges *g* [28]. Test–retest reliabilities (intraclass correlations; ICC) for $\dot{V}$O_2_max, Wingate, 1RM squat, CMJ, lower body force–velocity, and DXA were 0.95, 0.97, 0.90, 0.90, 0.97, and 0.99, respectively.

## 3. Results

### 3.1. Aerobic Capacity

**Figure 6:**
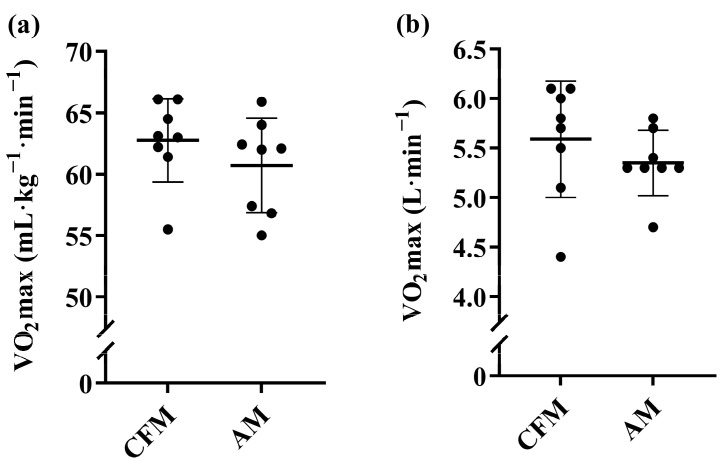
**Maximum oxygen consumption ($\dot{V}$O_2_max) for CrossFit men (CFM) and Alpinist men (AM).** N = 8 for CFM. N = 8 for AM. (**a**) Relative $\dot{V}$O_2_max. (**b**) Absolute $\dot{V}$O_2_max. Data are presented as mean ± SD, in addition to individual data points.

**Figure 7:**
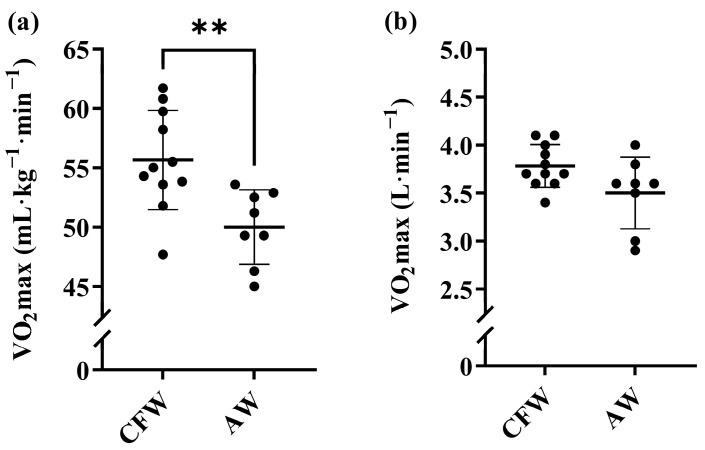
**Maximum oxygen consumption ($\dot{V}$O_2_max) for CrossFit women (CFW) and Alpinist women (AW).** N = 11 for CFW. N = 8 for AW. (**a**) Relative $\dot{V}$O2max. (**b**) Absolute $\dot{V}$O2max. Data are presented as mean ± SD, in addition to individual data points. ** Significant difference between groups (*p* < 0.01).

Relative and absolute values for $\dot{V}$O_2_max in men are presented in Figure 6. CFM had a $\dot{V}$O_2_max of 62.7 ± 3.4 mL·kg^−1^·min^−1^ and 5.6 ± 0.6 L·min^−1^, respectively. AM had a $\dot{V}$O_2_max of 60.7 ± 3.8 mL·kg^−1^·min^−1^ and 5.4 ± 0.3 L·min^−1^, respectively. There were no significant differences in $\dot{V}$O_2_max between CFM and AM in neither relative (mean ∆ = 3.4%, *p* = 0.279, ES = 0.53) nor absolute values (mean ∆ = 4.0%, *p* = 0.335, ES = 0.47). Relative and absolute values for $\dot{V}$O_2_max in women are presented in Figure 7. CFW had a $\dot{V}$O_2_max of 55.7 ± 4.2 mL·kg^−1^·min^−1^ and 3.8 ± 0.2 L·min^−1^, respectively. AW had a $\dot{V}$O_2_max of 50.0 ± 3.1 mL·kg^−1^·min^−1^ and 3.5 ± 0.5 L·min^−1^, respectively. There was a significant difference in $\dot{V}$O_2_max between CFW and AW in relative values (mean ∆ = 11.3%, *p* = 0.005, ES = 1.43). No difference in $\dot{V}$O_2_max was found in absolute values (mean ∆ = 8.3%, *p* = 0.055, ES = 0.91).

### 3.2. Anaerobic Capacity

Variables for anaerobic capacity are presented in Table 2. There were significantly higher values for CFM as compared to AM with regard to mean power (mean ∆ = 15.6%, *p* = 0.021, ES = 1.36) and relative mean power (mean ∆ = 14.2%, *p* = 0.018, ES 1.40). The same was true for CFW as compared to AW, showing higher values for mean power (mean ∆ = 13.0%, *p* = 0.008, ES = 1.41) and relative mean power (mean ∆ = 15.6%, *p* = 0.001, ES = 1.79) in the former group.

### 3.3. Squat and Jump Performance

Variables for squat performance and jump performance are presented in Table 3. There was no significant difference between CFM and AM nor between CFW and AW in the 1RM squat (mean ∆ = 5.4%, *p* = 0.272, ES = 0.50 and mean ∆ = −0.5%, *p* = 0.822, ES = 0.09, respectively), relative squat (mean ∆ = 4.7%, *p* = 0.737, ES = 0.39 and mean ∆ = 1.9%, *p* = 0.395, ES = 0.34, respectively), and CMJ jump height (mean ∆ = 5.6%, *p* = 0.920, ES = 0.46 and mean ∆ = 6.9%, *p* = 0.221, ES = 0.58, respectively).

### 3.4. Lower Body Force–Velocity

Variables for lower body force, velocity, and power are presented in Table 4. There were significantly higher values for CFM as compared to AM with regard to V_max_ (mean ∆ = 8.0%, *p* = 0.038, ES = 1.12), P_max_ (mean ∆ = 24.9%, *p* = 0.009, ES = 1.49), and relative P_max_ (mean ∆ = 24.0%, *p* = 0.003, ES = 1.76). The opposite was true for F_max_ (mean ∆ = −15.7%, *p* = 0.043, ES = 1.09) and relative F_max_ (mean ∆ = −16.2%, *p* = 0.034, ES = 1.31). There were significantly higher values for CFW as compared to AW with regard to P_max_ (mean ∆ = 22.8%, *p* = 0.003, ES = 1.58) and relative P_max_ (mean ∆ = 24.5%, *p* = 0.001, ES = 1.81). The opposite was true for F_max_ (mean ∆ = −14.1%, *p* = 0.034, ES = 0.61).

### 3.5. Body Composition (DXA)

Variables for body composition are presented in Table 5. CFM had a significantly lower fat mass and fat percentage as compared to AM (mean ∆ = −33.3%, *p* = 0.018, ES = 1.36 and mean ∆ = −27.4%, *p* = 0.027, ES = 1.16, respectively). The same was true for CFW as compared to AW, but the difference was significantly greater (mean ∆ = −37.5%, *p* = 0.002, ES = 1.67 and mean ∆ = −33.9%, *p* < 0.001, ES = 2.19, respectively). Lastly, CFW showed significantly higher values for the fat-free mass index (FFMI) as compared to AW (mean ∆ = 7.2%, *p* < 0.001, ES = 2.15).

## 4. Discussion

The purpose of this study was to map the physical and physiological characteristics of some of the best CrossFit athletes in the world, both men and women. In addition, to put the test results into perspective, they were compared to those of the Norwegian National team Alpinists. The main findings of this study were that the CrossFit athletes possess unprecedented physical capacities measured as $\dot{V}$O_2_max, anaerobic capacity (Wingate), as well as maximum strength and power in the lower limbs. Female CrossFit athletes had significantly higher aerobic capacities than their counterparts from the Alpinists, while no difference was found between the males. The CrossFit athletes of both sexes displayed higher anaerobic capacity and leg press power compared to the Alpinists. Finally, the key distinction was that the CrossFit athletes had lower fat mass and fat percentage than the Alpinists.

Although previous studies have attempted to illuminate the importance of $\dot{V}$O_2_max for performance in CrossFit, no studies have demonstrated as high relative values as in the current study for neither men nor women [4,7,8,9,10,11,16]. The highest average value for men seen so far is 58.4 ± 7.9 mL·kg^−1^·min^−1^ [7]. For the female CrossFit athletes, an average of ~44 mL·kg^−1^·min^−1^ has been reported [10], which is about 20% lower than in the CrossFit females in this study. Specifically, there were three CrossFit females that were >60 mL·kg^−1^·min^−1^ and with absolute values > 4 L·min^−1^. These absolute values (L·min^−1^) are on par with the best endurance athletes in the world, including rowing and cross-country skiing athletes [29]. This corresponds with the fact that these female CrossFit athletes are world-class athletes, and the CrossFit sport demands a large absolute work capacity, i.e., repeatedly lifting and carrying heavy external objects in short succession.

The $\dot{V}$O_2_max values from the Norwegian National team Alpinists were similar to what previous studies have reported from Alpinists with medals and experience from World Championships, in which values of 55–70 and 52–57 mL·kg^−1^·min^−1^ were found for men and women, respectively [19,30,31,32]. The sex difference in $\dot{V}$O_2_max values in this study was within the expected range: 12% for CrossFit and 18% for Alpinists [33].

The difference in relative $\dot{V}$O_2_max values between female CrossFit athletes and Alpinists may be explained by the difference in body composition. The Alpinists are heavier due to a significantly higher fat mass and body fat percentage as compared to the CrossFit athletes (further discussed below). It is known that $\dot{V}$O_2_max scaled to body size may not raise with a power of 1, which may explain the lower values observed for the Alpinists [34].

The overall Wingate results of both CrossFit athletes and Alpinists in the current study can be described as “elite” based on reported mean power values from a large selection of athletes [29]. This is not surprising, as anaerobic power is a defining property in both sports [6,8,9,10,19,20]. The positive associations previously reported between anaerobic capacity and performance are intuitively sound as many CrossFit workouts typically include short high-intensity exercises, such as biking and rowing sprints [6,8,9,10]. Feito and coworkers tested the Wingate performance of 29 advanced CrossFit athletes (15 men and 14 women) [6]. For the men, they found similar results for both absolute and relative peak power output (1545 ± 230 W and 17.1 ± 2.6 W·kg^−1^, respectively), while mean power output values were more than 20% lower than in the present study. For the women, similar differences were seen. Slight differences in the mass-adjusted workload for the Wingate protocol (men: 8.5% and women: 8.2% in Feito’s study) may have affected the results, but apparently, the athletes in the present study were considerably more fatigue-resistant than those in the study by Feito and coworkers [6]. Of note, in the current study, the Wingate test was performed seated, which results in lower peak values than if standing [35]. It is unclear if Feito and coworkers allowed a standing sprint.

A limitation of the current study was that the Wingate test for the CrossFit athletes was carried out on the Lode Excalibur, whereas the Alpinists used the Wattbike. As mentioned before, ergometer bikes can calculate peak values in different ways. Lode Excalibur records values every 200 ms, whereas the Wattbike registers per pedal cycle. This means the Alpinists may have demonstrated slightly higher peak power values if they had been tested on the Lode Excalibur bike, while this should not have affected the mean power values.

Previous studies have reported somewhat lower or similar 1RM squat values in CrossFit-trained men, while the values were more than 30% lower for the women compared to the current study [5,10]. The 1RM values were similar between the CrossFit athletes and the Alpinists. A 1RM of 112 ± 11 kg has previously been reported in elite female Alpinists [36], which is more than 10% lower than in the present study.

Both relative and absolute maximum strength in the squat has been shown to be related to CrossFit performance [4,5,7,8,10,11,12,13]. This could be explained by the fact that 1RM squat performance is strongly correlated to greater strength in the snatch and clean and jerk [37], as well as sprinting and jumping [38], which are all exercises frequently found in CrossFit workouts [1].

CMJ performance has been previously investigated in male CrossFit athletes. However, these studies report values that are about 20% lower compared to the current study [8,14]. Just as with the squat, CMJ performance has been shown to correlate with CrossFit performance [8,14].

The overall results of both the CrossFit athletes and Alpinists in the current study match elite-level CMJ heights based on a large selection of national team athletes, which reported values of 45 ± 6 and 36 ± 5 cm for male and female Alpinists, respectively [39].

It is worth mentioning that a HUR labs portable power platform was used during testing of the CrossFit athletes, while the Alpinists jumped on a floor-mounted AMTI power platform. Unpublished data suggest that the difference between these methods is 5–10%, with HUR showing higher values. This means that the comparison between CrossFit and Alpine skiing must be interpreted with caution.

Compared to previous studies that have investigated leg press force and power, the participants in the current study showed results on par with competitive athletes (F_max_: ~3750 ± 800 N, P_max_: ~2850 ± 750 W) [25]. This is supported by unpublished data that clearly show that the Alpinists are among the most powerful athletes in Norway—including weightlifters, powerlifters, and track and field athletes.

The fact that the Alpinists developed more force (N) in the leg press test than the CrossFit athletes may be explained by the requirement of handling greater forces than what CrossFit athletes have to handle. Due to the high loads and shocks that occur during turns and landings in races for Alpinists, high demands are placed on concentric, eccentric, and isometric strength [40,41]. It has previously been documented that Alpinists must withstand a ground reaction force of >3 × body weight [42]. The discrepancy with the 1RM squat (similar values) may be explained by the seated leg press position, which resembles a skiing-specific position; and/or the CrossFit athletes are technically more skilled in the squat exercise than the Alpinists.

One explanation for the CrossFit athletes’ higher power (W) may be due to the regular implementation of Olympic weightlifting [1]. As is well known, Olympic weightlifting is strongly related to the development of power [43]. Despite this difference, the implementation of Olympic weightlifting is not unusual for Alpinists during periods were they want to develop power [40].

Both male and female CrossFit athletes had a significantly lower body fat percentage and fat mass as compared to the Alpinists. The difference between the women, however, was greater than between the men. Mangine and coworkers previously measured the body composition of eight (4 men and 4 women) advanced CrossFit athletes [16]. In this respective study, the men showed a similar body fat percentage at 11 ± 3%, while the women showed a lower body fat percentage at 12 ± 2%. Of note, these body fat percentages were estimated using a 4-compartment model [16]. The body fat percentage for the Alpinists in the current study showed only slightly higher values than those reported earlier [19,31,32]. It must be considered that in the current study, DXA was used to determine the body composition, which typically shows higher fat mass values as opposed to other methods, such as skinfold measurements [44,45].

Body composition is an important factor in sports performance [46], but its practical implications are different for CrossFit and Alpine skiing. The body composition difference is consistent with the demands of the sports. In CrossFit, a lower body fat percentage has been shown to be significantly correlated with better performance [13]. CrossFit athletes depend on optimal body composition to perform in bodyweight exercises (calisthenics) such as pull ups, muscle ups, handstand walks, etc. These movements require horizontal or vertical displacement of body mass, in which unnecessary fat mass will increase the energy requirement and thus negatively impact exercise efficiency [47]. For Alpinists, on the other hand, it is conceivable that slightly more fat mass will have a positive effect on performance in the speed disciplines super-G and downhill [48].

The most striking feature of the female CrossFit athletes in the current study is the amount of fat-free mass in relation to body height, as indicated by the FFMI. With an FFMI of 20.5 kg·m^−2^, the female CrossFit athletes in the current study are well above the average of 17.6 kg·m^−2^ found in a study of a large cohort of Olympic athletes [49]. Further studies should aim to investigate whether this large muscle mass is a genetic prerequisite or whether CrossFit training efficiently stimulates athletes to gain the necessary muscle mass.

There are several limitations in the current study. As previously mentioned, there were some differences in equipment used to test the CrossFit athletes and Alpinists. In addition, the variation in the personnel performing the measurements could have introduced errors. No upper body tests were performed in this study, which could have further specified the physiological characteristics of the CrossFit athletes. Lastly, as this was a cross-sectional study, nothing certain can be said about cause and effect.

### Practical Applications

The current study reveals that CrossFit athletes at the highest level are truly elite athletes who possess physical and physiological characteristics comparable to other world-class athletes. These findings offer valuable insights into the physical demands of elite-level CrossFit, allowing for better identification of talent and sports success by practitioners. Since CrossFit encompasses a wide range of physical challenges, from endurance tasks like long-duration workouts to strength-based exercises like heavy lifting, there can be a large variety between competitors based on their individual strengths, weaknesses, and training focus. Recognizing this variability is key when determining the ideal training regimen and competition strategy for each athlete. Coaches could use the cross-sectional data from this study to identify the strengths and weaknesses of their athletes and adjust their respective training programs to meet these elite-level standards. Moreover, the current study introduces a comprehensive testing protocol for assessing athlete progress and identifying areas for improvement. Researchers can leverage these findings to formulate hypotheses for longitudinal training studies.

## 5. Conclusions

CrossFit is a modern sport that has grown into a worldwide sport. In this study, the physical and physiological characteristics of Norwegian international-level CrossFit athletes were profiled against the backdrop of truly elite Alpinists: the Norwegian National team. The current observations show that elite CrossFit athletes are very fit across both aerobic and anaerobic capacities and possess high maximum strength and power while maintaining exceptional body composition—comparable to world-class Alpinists and in correspondence with the all-round fitness idea of CrossFit. Further studies are warranted for a better understanding of these characteristics in relation to competition performance at the elite level.

## Figures and Tables

**Table 1:** Physical characteristics of the participants.

	CFM (*n* = 8)	AM (*n* = 8)	CFW (*n* = 11)	AW (*n* = 8)
**Age (yrs)**	28.4 ± 3.0	26.5 ± 5.7	29.2 ± 4.4	25.6 ± 3.5
**Height (m)**	181.1 ± 7.2	183.5 ± 3.5	168.4 ± 5.1	170.1 ± 2.9
**Weight (kg)** **BMI (kg·m^−2^)**	88.5 ± 7.7 26.9 ± 0.9	90.0 ± 8.3 26.7 ± 2.0	67.9 ± 5.3 23.9 ± 0.8	69.7 ± 5.3 24.1 ± 1.5

Data are presented as mean ± SD. Abbreviations; CFM: CrossFit men. AM: Alpinist men. CFW: CrossFit women. AW: Alpinist women. BMI: Body Mass Index.

**Table 2:** Anaerobic capacity.

	CFM (*n* = 8)	AM (*n* = 7)	*p*-Value	ES	CFW (*n* = 9)	AW (*n* = 8)	*p*-Value	ES
**Peak power (W)**	1497 ± 170	1398 ± 181	0.296	0.56	925 ± 121	1040 ± 123	0.149	0.90
**Relative peak power (W·kg^−1^)**	16.8 ± 1.3	15.8 ± 1.6	0.234	0.66	13.6 ± 1.5	14.9 ± 1.4	0.088	0.84
**Mean power (W)**	968 ± 90	837 ± 103	0.021	1.36	644 ± 51	570 ± 48	0.008	1.41
**Relative mean power (W·kg^−1^)**	10.9 ± 0.8	9.5 ± 1.2	0.018	1.40	9.5 ± 0.6	8.2 ± 0.8	0.001	1.79

Data are presented as mean ± SD. Abbreviations; CFM: CrossFit men. AM: Alpinist men. CFW: CrossFit women. AW: Alpinist women. ES = Effect size.

**Table 3:** Squat and jump performance.

	CFM (*n* = 8)	AM (*n* = 8)	*p*-Value	ES	CFW (*n* = 11)	AW (*n* = 8)	*p*-Value	ES
**1RM squat (kg)**	188.2 ± 14.8	178.6 ± 21.4 *	0.324	0.50	130.6 ± 7.2	131.3 ± 7.6	0.849	0.09
**Relative squat (1RM·kg^−1^)**	2.13 ± 0.19	2.03 ± 0.28 *	0.439	0.39	1.93 ± 0.16	1.88 ± 0.14	0.460	0.34
**CMJ jump height (cm)**	50.2 ± 2.5	47.5 ± 7.2	0.353	0.46	39.6 ± 4.7 ^#^	37.0 ± 3.6	0.221	0.58

Data are presented as mean ± SD. Abbreviations; CFM: CrossFit men. AM: Alpinist men. CFW: CrossFit women. AW: Alpinist women. ES = Effect size. 1RM: 1 repetition maximum. CMJ: countermovement jump. * *n* = 7. ^#^ *n* = 10.

**Table 4:** Lower body force–velocity.

	CFM (*n* = 8)	AM (*n* = 7)	*p*-Value	ES	CFW (*n* = 10)	AW (*n* = 8)	*p*-Value	ES
**F_max_ (N)**	3398 ± 426	4028 ± 656	0.043	1.09	2288 ± 160	2701 ± 440	0.034	1.25
**V_max_ (m·s^−1^)**	5.3 ± 0.4	4.8 ± 0.3	0.038	1.12	4.5 ± 0.2	4.7 ± 0.4	0.198	0.61
**P_max_ (W)**	3020 ± 388	2418 ± 371	0.009	1.49	1923 ± 231	1566 ± 193	0.003	1.58
**Relative F_max_ (N·kg^−1^)**	38.4 ± 3.2	45.8 ± 7.1	0.034	1.31	33.5 ± 2.3	39.0 ± 7.7	0.088	0.97
**Relative P_max_ (W·kg^−1^)**	34.1 ± 2.9	27.5 ± 4.1	0.003	1.76	28.1 ± 2.6	22.6 ± 3.3	0.001	1.81

Data are presented as mean ± SD. Abbreviations; CFM: CrossFit men. AM: Alpinist men. CFW: CrossFit women. AW: Alpinist women. ES = Effect size. F_max_: maximum force. V_max_: maximum velocity. P_max_: maximum power.

**Table 5:** Body composition.

	CFM (*n* = 8)	AM (*n* = 8)	*p*-Value	ES	CFW (*n* = 9)	AW (*n* = 8)	*p*-Value	ES
**Fat mass (kg)**	10.0 ± 1.9	15.0 ± 4.6	0.018	1.36	10.2 ± 2.2	16.3 ± 4.5	0.002	1.67
**Fat-free mass (kg)** **Lean mass (kg)**	79.3 ± 7.4 75.3 ± 7.0	79.4 ± 4.6 75.5 ± 4.5	0.971 0.957	0.02 0.03	58.0 ± 3.4 55.1 ± 3.3	55.3 ± 2.9 52.3 ± 2.8	0.100 0.085	0.81 0.85
**Bone mass (kg)**	4.0 ± 0.6	3.9 ± 0.2	0.637	0.23	3.0 ± 0.3	2.9 ± 0.2	0.730	0.16
**Fat % (-)**	11.8 ± 2.4	16.2 ± 4.5	0.027	1.16	15.5 ± 2.3	23.5 ± 4.4	<0.001	2.19
**FFMI (kg·m^−2^)**	24.1 ± 0.9	23.6 ± 1.4	0.384	0.43	20.5 ± 0.5	19.1 ± 0.7	<0.001	2.15

Data are presented as mean ± SD. Abbreviations; CFM: CrossFit men. AM: Alpinist men. CFW: CrossFit women. AW: Alpinist women. ES = Effect size. Fat %: fat percentage. FFMI: fat-free mass index.

## Data Availability

The raw data supporting the conclusions of this article will be made available by the authors on request.
